# Creutzfeldt-Jakob Disease: A Case Report and Literature Review for Understanding the Big Picture

**DOI:** 10.7759/cureus.31303

**Published:** 2022-11-09

**Authors:** Shubhangi Barnwal, Gaurav Jha, Siri Chandana Sola, Preethu Anand, Sameer Y Shariff

**Affiliations:** 1 Infectious Diseases, Leicester Royal Infirmary, Leicester, GBR; 2 Orthopaedics, Leicester Royal Infirmary, Leicester, GBR; 3 Cardiology, Narayana Hrudayalaya, Bangalore, IND; 4 Neurology/Stroke Medicine, Queen's Hospital, London, GBR; 5 Internal Medicine, University Hospitals of Leicester NHS Trust, Leicester, GBR; 6 Internal Medicine, University Hospitals of Leicester NHS Trust - Leicester Royal Infirmary, Leicester, GBR; 7 General Medicine, University Hospitals of Leicester NHS Trust, Leicester, GBR

**Keywords:** prion disease, rare neurodegenerative disease, high fatality, rapidly progressive dementia, creutzfeldt-jakob disease

## Abstract

Creutzfeldt-Jakob disease (CJD) is a rare, rapidly progressive neurodegenerative disorder that has an invariably fatal outcome. Aside from rapidly progressive dementia, this condition manifests as myoclonus, cerebellar, pyramidal, extrapyramidal, visual, and psychiatric symptoms. On the other hand, nonspecific symptoms might be difficult to diagnose, leading to a late or incorrect diagnosis. Given its high fatality, most patients die within 12 months of the disease's onset.

This case report describes a healthy man who presented with cerebellar and pyramidal signs along with memory loss worsening over six weeks. He also had indications of corticobasal degeneration, such as myoclonus and alien limb syndrome, but with reasonably maintained cognition. These signs are all non-specific, and premortem diagnosis is typically difficult and challenging owing to a lack of knowledge and clinical suspicion. However, after a thorough and extensive investigation, CJD was diagnosed.

Despite the fact that CJD is a rare disease, it should always be included in the differential diagnosis whenever neuropsychological manifestations are present. Nevertheless, CJD can be successfully and promptly ruled out with a detailed clinical examination and appropriate investigation.

## Introduction

Classic CJD is a life-threatening, neurodegenerative disorder that occurs in humans as well as a wide variety of animals with characteristic clinical and diagnostic features. It is thought to be brought on by replicating an aberrant isoform of encoded proteins called prion protein [1]. This disease's most typical early symptom is cognitive impairment, which can be seen in 35% of patients. Cerebellar dysfunction, behavioral symptoms, or constitutional symptoms might potentially be the first sign in about 17.5% of cases [2]. There are four different forms of CJD that have been identified so far: familial, iatrogenic, variant, and sporadic. The sporadic form of the disease is the most frequent and accounts for 85% of CJD cases [2,3].

The disease has a short course and an average onset of 65 years, with most cases falling between the ages of 60 and 80 [3]. Sporadic Creutzfeldt-Jakob disease (sCJD) has an unknown aetiology with a median survival of 3.5-6 months with an incidence reported at 1-1.5 per million [4,5]. It results in a subacute and progressive deterioration in cognitive, motor and behavioural function over a period of weeks to months. CJD is characterised by spongiform alteration, neuronal death, and gliosis as a result of aberrant prion protein buildup in the brain [6].

The clinical features of CJD are varied and include both neurological and psychological symptoms. Due to its variable presentation, it poses a diagnostic challenge. The diagnosis of CJD is frequently suspected on the basis of clinical presentation, investigation and imaging studies like magnetic resonance imaging (MRI), electroencephalogram (EEG) and cerebrospinal fluid (CSF) analysis. However, the confirmatory diagnosis of CJD requires neuropathologic and immunodiagnostic testing of brain tissue obtained from either a biopsy or autopsy [6].

## Case presentation

A previously healthy 57-year-old man presented to the emergency department (ED) with a two-week history of slurred speech, vertigo, worsening imbalance while walking, and memory loss. The patient's family reported that he had a severe, sudden onset of headache a few weeks ago, followed by progressive numbness in his hands, feet and forehead and that he also had new onset memory disturbances on occasions such as forgetting his signature, word-finding difficulties and reciting prayer lines. It's interesting to note that he had seen his general practitioner (GP) a week prior to his ED attendance for similar concerns when he was prescribed prochlorperazine and sent home without any investigation of his neurology. Nevertheless, a couple of days later, his symptoms took a turn for the worse and his slurring of speech got worsened. He denied any other symptoms. He had no significant past medical history, family history or psychiatric history of note. He was a non-smoker and denied any alcohol or recreational drug use.

His initial general physical examination was unremarkable. However, neurological examination revealed slurred speech, slight past pointing and gait ataxia. All other systemic examinations were normal. An initial impression of stroke was ruled out on computerised tomography (CT) head scan, which showed no acute intracranial findings. An electrocardiogram (ECG) demonstrated normal sinus rhythm and an urgent outpatient magnetic resonance Imaging (MRI) head scan was requested in view of abnormal neurological findings. Blood tests, including full blood count, liver function tests, renal function tests, and C-reactive protein, were normal.

**Figure 1:**
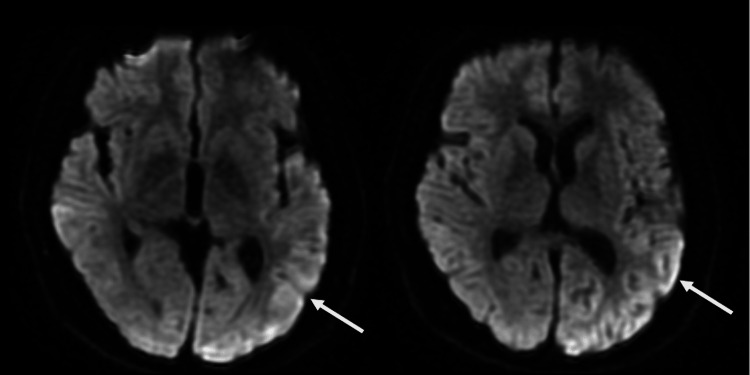
MRI diffusion-weighted images showing hyperintense signal in the cingulate gyrus

**Figure 2:**
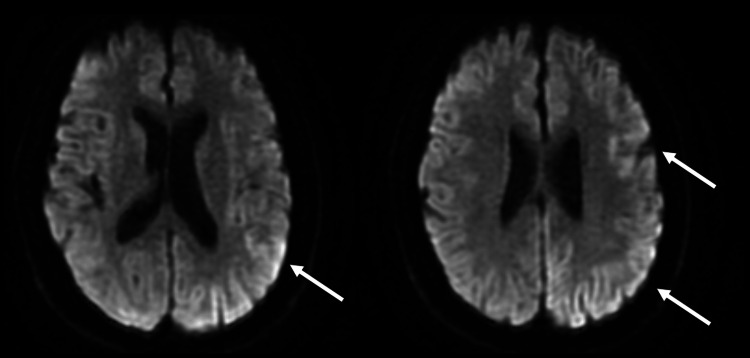
MRI diffusion-weighted images showing a hyperintense signal in the temporo-parietal lobe

**Figure 3:**
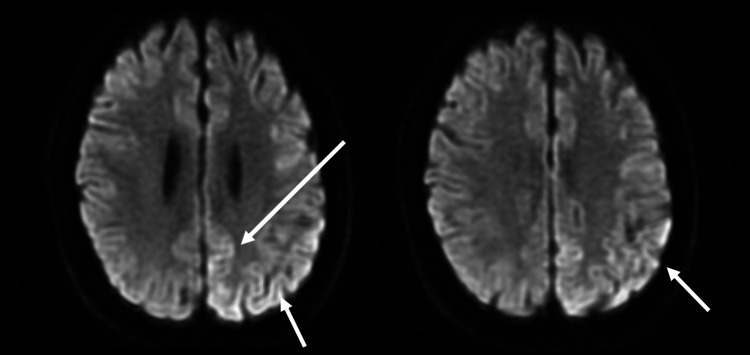
MRI diffusion-weighted images showing a hyperintense signal in the left temporal cortex region

**Figure 4:**
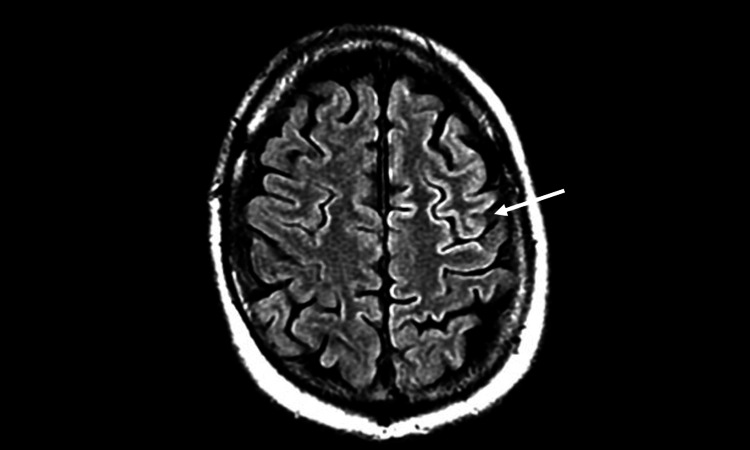
Increased T2 FLAIR signal changes in the frontal lobe FLAIR: fluid-attenuated inversion recovery

The outpatient MRI head showed extensive cortical diffusion restriction mainly affecting the left frontal lobe and cingulate gyrus on diffusion-weighted imaging (DWI). A differential diagnosis of CJD and seizure-related changes were considered. A subsequent MRI head with contrast imaging was requested, which showed constant areas of altered signal changes on DWI and apparent diffusion coefficient (ADC), and a corresponding increased fluid-attenuated inversion recovery (FLAIR) signal of the cortex in the left fronto-temporal-parietal lobes without pathological contrast enhancement, suspicious of Creutzfeldt-Jakob disease (Figures 1-4).

On admission, he had multidirectional nystagmus, severe gait ataxia and significant bilateral dysmetria. He also complained of a classical alien limb phenomenon with the involuntary motor activity of his right upper limb. All other neurological examinations were normal. An extensive blood workup was done, which included vitamin E, human immunodeficiency virus (HIV) and syphilis serology, erythrocyte sedimentation rate (ESR), rapid plasma reagin (RPR), hepatitis B & C, antibodies screening, vasculitis screening, and celiac screening infection panel came back negative.

An EEG showed left hemispheric dysfunction with epileptogenicity: intermittent slow delta wave activity and sharp and slow wave complexes frequently seen repetitively, semi-periodically (periodic lateralized epileptiform discharges) over the left hemisphere, maximal over the left posterior quadrant. Lumbar puncture showed a cerebrospinal fluid band with normal protein, glucose and cell count. Two weeks after his initial presentation, the patient complained of reduced visual acuity for the first time following which he was reviewed by the ophthalmology team. The ocular examination was normal. MRI orbit revealed progression of the cortical diffusion restriction primarily affecting the left cerebral hemisphere.

The patient was started on levetiracetam because of a new onset of seizures. Based on the clinical symptoms and imaging manifestations, the possibility of paraneoplastic syndrome or subacute cerebellar syndrome or CJD was considered. To rule out malignancy, CT chest, abdomen, and pelvis (CAP) was done, which showed a prominent and significant soft tissue growth at the ileocecal valve, for which colonoscopy was advised. A further outpatient positron emission tomography (PET)-CT scan was also planned. With a rapidly progressive neurological syndrome within six weeks, pre-dominant pyramidal signs, cerebellar and corticobasal syndrome, myoclonus, alien limb syndrome, dysarthria and relatively preserved cognition along with positive findings on MRI head paved the clinical diagnosis of CJD.

The possibility of the paraneoplastic syndrome was incredibly unlikely. The multidisciplinary team decided not to investigate his condition any further and to discharge him with a package of care due to his guarded prognosis. However, the patient's condition worsened because of symptoms including a new onset of hallucinations, increased aggression, involuntary jerky movements, visual loss, insomnia and altered behaviour. He became more bed-bound. as his mobility declined.

The patient's general condition had rapidly deteriorated during the previous eight weeks. He was examined by the palliative care staff, prescribed pro re nata (PRN) anticipatory drugs and sent to the hospice where he was kept comfortable and passed away while at home after a couple of months.

## Discussion

The clinical features of CJD are varied and include both neurological and psychological symptoms. Due to its variable presentation and lack of knowledge, it is often difficult and poses a challenge for a premortem diagnosis.

The initial neurological symptoms of sporadic CJD can include cerebellar and corticobasal syndrome along with vision problems, which may progress to blindness in advanced cases. Muscle weakness with loss of muscle mass, twitches and shock-like spasms (myoclonus) are also seen. It also can include urinary incontinence, bowel incontinence and dysphagia. On the other hand, initial psychological symptoms of variant CJD can include severe depression, anxiety, irritability and difficulty sleeping (insomnia), which gradually progress into loss of memory, problem concentrating, confusion, feeling agitated, aggressive behaviour, loss of appetite resulting in weight loss, paranoia, unusual and inappropriate emotional responses.

A series of investigations like periodic sharp wave complex on EEG, 14-3-3 protein in the CSF and aberrant signal changes in caudate nuclei and putamen on DWI or FLAIR MRI can all be used to support the diagnosis of sCJD. However, a conclusive diagnosis of sCJD can only be made by confirming pathologic prion protein deposition in the brain usually done at autopsy or seldom on a brain biopsy [7].

The pathophysiology of CJD is believed to be caused by defective proteins that steadily accumulate in the brain cells (neurons), causing eventual damage and neuronal death. Prions change the structure of normal proteins in the brain to fold abnormally and further trigger the production of more prion protein, which is a key event in prion disease pathology. This conversion occurs at a rapid rate, which explains why CJD symptoms can vary from mild to severe in a short span of time. People with all kinds of CJD will become entirely bedridden when the disease enters its last stages. They frequently lose all awareness of their surroundings and need 24-hour care.

Considering Centers for Disease Control and Prevention (CDC) criteria for CJD (Table 1), the clinical presentation and the progression of the disease over weeks, this case was that of a sporadic CJD, with key findings of rapidly progressive decline in cognition, slurred speech, memory loss and balance problems, and abnormal MRI and EEG findings [6].

**Table 1:** 2010 Centers for Disease Control and Prevention (CDC) criteria for sporadic CJD CJD: Creutzfeldt-Jakob disease

Clinical Diagnostic Criteria for Sporadic CJD
Definitive Diagnosis – Detection of protease-resistant prion protein or scrapie-associated fibrils by neuropathology, immunochemical technique and/or western blot.
Probable Diagnosis – Neuropsychiatric symptoms along with a positive RT- QuIC in cerebrospinal fluid (CSF) or other tissues. OR No findings indicating alternative diagnoses and progressive dementia with at least 2 of (1) – (4) and at least one of (a) – (c).
Possible Diagnosis – No routine investigations or findings indicating alternative diagnoses and progressive dementia with a duration of less than 2 years and with at least 2 of (1) – (4) and the absence of any positive result that would classify the case as ‘probable’.
Myoclonus Visual or cerebellar symptoms Pyramidal or extrapyramidal features Akinetic mutism
Periodic sharp wave complexed on EEG Positive 14-3-3 protein in the cerebrospinal fluid with a disease duration of less than 2 years. High signal abnormalities in caudate nucleus and putamen on diffusion-weighted imaging (DWI) or fluid-attenuated inversion recovery (FLAIR) MRI.

The term "familial CJD" refers to definite or probable CJD along with a definite or probable CJD in a first-degree relative, whereas a progressive cerebellar syndrome in a person who received pituitary hormone sourced from human cadavers, or sporadic CJD with a known exposure risk, are both considered forms of iatrogenic CJD (receiving treatment for short stature, or dura mater transplant) [8]. The first description of variant CJD (vCJD) was made in the UK in 1996. It is a zoonotic type of human prion disease that was brought about by dietary contamination of human food with substances from animals that had the disease bovine spongiform encephalopathy (BSE), commonly called ‘mad cow' disease [9].

When it comes to cognitive decline in CJD patients, it is commonly misdiagnosed as frontotemporal dementia, dementia with Lewy bodies or quickly developing Alzheimer's disease. Ataxic manifestations are frequently linked to acquired CJD and could be classified as vascular, neoplastic, paraneoplastic or inflammatory illnesses that affect the cerebellum and its connections to the brainstem.

Diagnostic indicators

One of the diagnostic indicators of CJD is the cortical ribbon sign (hyperintensity on the cortical gyri on DWI and FLAIR sequences), which was seen in this patient. It will assist us in eliminating other types of variance. Most sCJD patients have cerebral atrophy or an increase in signal intensity in the cerebral cortex, putamen and caudate nucleus (particularly on a DWI sequence). Regarding the other types of CJD, familial CJD and variant CJD are both characterised by basal ganglia and cortical hyperintensity, respectively [10]. Recent research has demonstrated that utilising DWI and FLAIR sequences, MRI can reliably identify sCJD-specific alterations with 91% sensitivity and 95% specificity. This patient got MRI contrast with DWI and FLAIR to generate a more accurate image because studies have shown that DWI has higher sensitivity than FLAIR. However, because MRI is a non-invasive diagnostic, it should be performed on all individuals who show indications of CJD [11].

EEG has been used therapeutically to differentiate specific brain pathological diseases by measuring brain function. Periodic sharp wave complexes are found in 50% of sCJD patients, especially in the latter stages [8].

Recent advancement

The National Prion Disease Pathology Surveillance Centre created a new diagnostic test termed the second-generation real-time-quaking-induced conversion (RT-QuIC) in April 2015. Few studies have indicated that protein 14-3-3, neuron-specific enolase, and t-tau protein have better specificity (98%) for human prion disease and could be more sensitive than CSF utilising olfactory epithelium (from nasal brushings) [10]. According to a recent study, an adaptation of RT-QuIC, called e-QuIC, was developed, which can detect up to 10^14^-fold dilutions and has a sensitivity of 10^4^ times more than its predecessor [12]. RT-QuIC is less invasive than brain biopsy and can be used in place of brain biopsy to accurately diagnose CJD. It should be the first test conducted in the workup of a patient suspected of having CJD since it is less intrusive.

Clinical suspicion must be sufficiently strong or low to warrant a biopsy, and a negative biopsy does not rule out CJD since it can predominate in particular brain regions. A biopsy is therefore unlikely to be undertaken in either of these situations [13]. Although tissue testing remains the most definitive method of confirming CJD diagnosis, it should be noted that even a biopsy isn't always conclusive. Deposits of prion protein (scrapie) can be found in the skeletal muscle and spleen of one-third of people with sporadic CJD [14].

## Conclusions

CJD is indeed a rare disease with a poor prognosis and rapid progression. Nevertheless, any time neuropsychological signs, particularly a gradual decrease in cognition, are present in the patient together with symptoms like visual hallucinations, myoclonus and ataxia, it should be taken into consideration when making a differential diagnosis. This can aid in making an early and accurate diagnosis allowing patients and their families to an expected disease course. For CJD, there is no proven therapy and hence supportive and symptomatic care are the cornerstones of therapy. There have been a few medication studies on CJD, but none of them has so far clearly demonstrated a benefit. For this lethal illness to be treated, more study is required.
